# Two-Steps Versus One-Step Solidification Pathways of Binary Metallic Nanodroplets

**DOI:** 10.1021/acsnano.2c09741

**Published:** 2022-12-20

**Authors:** Diana Nelli, El Yakout El Koraychy, Manuella Cerbelaud, Benoit Crespin, Arnaud Videcoq, Alberto Giacomello, Riccardo Ferrando

**Affiliations:** † Dipartimento di Fisica, Università di Genova, Via Dodecaneso 33, 16146Genova, Italia; ‡ Université de Limoges, CNRS, IRCER, UMR 7315, F-87000Limoges, France; § Université de Limoges, CNRS, XLIM/ASALI, F-87000Limoges, France; ∥ Dipartimento di Ingegneria Meccanica e Aerospaziale, Sapienza Università di Roma, via Eudossiana 18, 00184Roma, Italia

**Keywords:** solidification, nanodroplets, simulations, silver, cobalt, nickel, copper

## Abstract

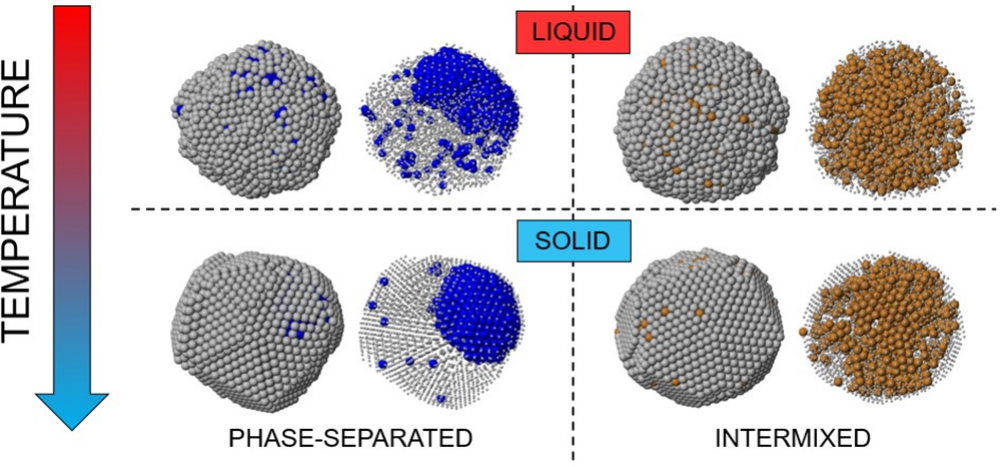

The solidification of AgCo, AgNi, and AgCu nanodroplets is studied by molecular dynamics simulations in the size range of 2–8 nm. All these systems tend to phase separate in the bulk solid with surface segregation of Ag. Despite these similarities, the simulations reveal clear differences in the solidification pathways. AgCo and AgNi already separate in the liquid phase, and they solidify in configurations close to equilibrium. They can show a two-step solidification process in which Co-/Ni-rich parts solidify at higher temperatures than the Ag-rich part. AgCu does not separate in the liquid and solidifies in one step, thereby remaining in a kinetically trapped state down to room temperature. The solidification mechanisms and the size dependence of the solidification temperatures are analyzed, finding qualitatively different behaviors in AgCo/AgNi compared to AgCu. These differences are rationalized by an analytical model.

## Introduction

Solidification processes are of paramount importance in many contexts concerning the production of materials, which are often initially prepared at high temperature in the liquid phase and then cooled to reach operating conditions. Nanoscale materials can be produced according to the same scheme.^1,2^ Despite their importance, solidification processes at the nanoscale have rarely been studied, and they are poorly understood. In particular, very little is known about the solidification of binary metallic nanoparticles. In these nanoparticles, the chemical ordering can be deeply influenced by the solidification process, which therefore becomes even more crucial for determining the properties relevant for applications than in single-element nanoparticles. Determining nanoscale solidification pathways at an experimental level is extremely difficult due to their short time scales and typically high temperatures. For this reason computer simulations can be of great help, because they reproduce the relevant mechanisms at the atomic level. Here we simulate the solidification of binary nanodroplets composed by phase-separating elements. We show that very different pathways can occur, depending on whether phase separation takes place already in the liquid phase or not. This key step determines the hallmarks of the subsequent evolution: approach to equilibrium versus kinetic trapping of chemical ordering; two-step versus one-step solidification process; heterogeneous nucleation versus homogeneous; stronger or weaker dependence of the solidification temperature on the nanoparticle size. These points are here addressed and discussed in detail. The size dependence of the temperature in one-step solidification is rationalized by an analytical approach based on nucleation theory^3^ and on a low-temperature expansion of the free energy barrier above the instability of the liquid.^4^

The freezing of liquid nanodroplets has been studied by simulations in several elemental metallic systems.^5−11^ On the other hand, there are fewer simulations of the freezing of binary metallic nanodroplets. The majority of these simulations focused on systems with a tendency to intermixing between the two elements, such as AgAu, AlNi, AuPd, and NiCo.^12−20^ Fewer studies were devoted to systems with a strong tendency toward phase separation. In particular, the freezing of AgCo, CuCo, CuNi, and AlFe nanodroplets was simulated^16,21,22^ and, more recently, also the freezing of AgCu and AgNi nanoalloys.^23−25^ The free-energy barrier for crystal nucleation in CuNi and CuPd nanoalloys has been calculated at fixed sizes and varying composition.^26,27^ Melting has been studied in several simulations that revealed the occurrence of a two-step process. In fact, phase-separating nanoalloys such as AgCu, CuNi, AgNi, AuCo, AuNi, AgCo, AuFe, and several others adopt core–shell and quasi-Janus structures in their solid state,^23,28−30^ and their shell can melt at a lower temperature than their core.^31−33^ On the contrary, the occurrence of single-step or two-step freezing processes is not yet studied.

In this paper we consider three systems of the type AgX, where X = Co, Ni, and Cu. All these systems are weakly miscible and are expected to form core–shell structures at the equilibrium, with Ag in the shell. Ag atoms tend to segregate to the nanoparticle surface because of the lower surface energy and larger atomic size of Ag compared to Cu, Co, and Ni. However, these three systems present some differences. In AgCo and AgNi the tendency to phase separation is rather extreme, with the miscibility gaps extending deep into the liquid phase,^34,35^ while AgCu presents a somewhat milder phase separation tendency.^36^

We consider the same composition for all systems, i.e., our nanoalloys always contain 75 atom % of Ag and 25 atom % of X. For this composition, the core is expected to be always covered completely by Ag atoms, for all nanoalloy sizes considered in the simulations. The size *N* of the nanoparticles varies from a minimum of 250 to a maximum of 10 000 atoms, i.e., with diameter *d* in the range from 2 to 8 nm. The solidification of these nanoparticles is studied by molecular dynamics (MD) simulations, which allow one to follow physical atomic trajectories, so that they can shed light on the key atomic-level mechanisms of this type of dynamical process. Simulations are performed by using a wide range of cooling rates, from 0.1 to 10 K/ns. The initial configurations are chosen at the liquid state at high temperature (see Methods section for details), then the nanodroplets are cooled down to room temperature.

## Results and Discussion

### Intermixing Versus Phase Separation in the Liquid and in the Solid

**Figure 1:**
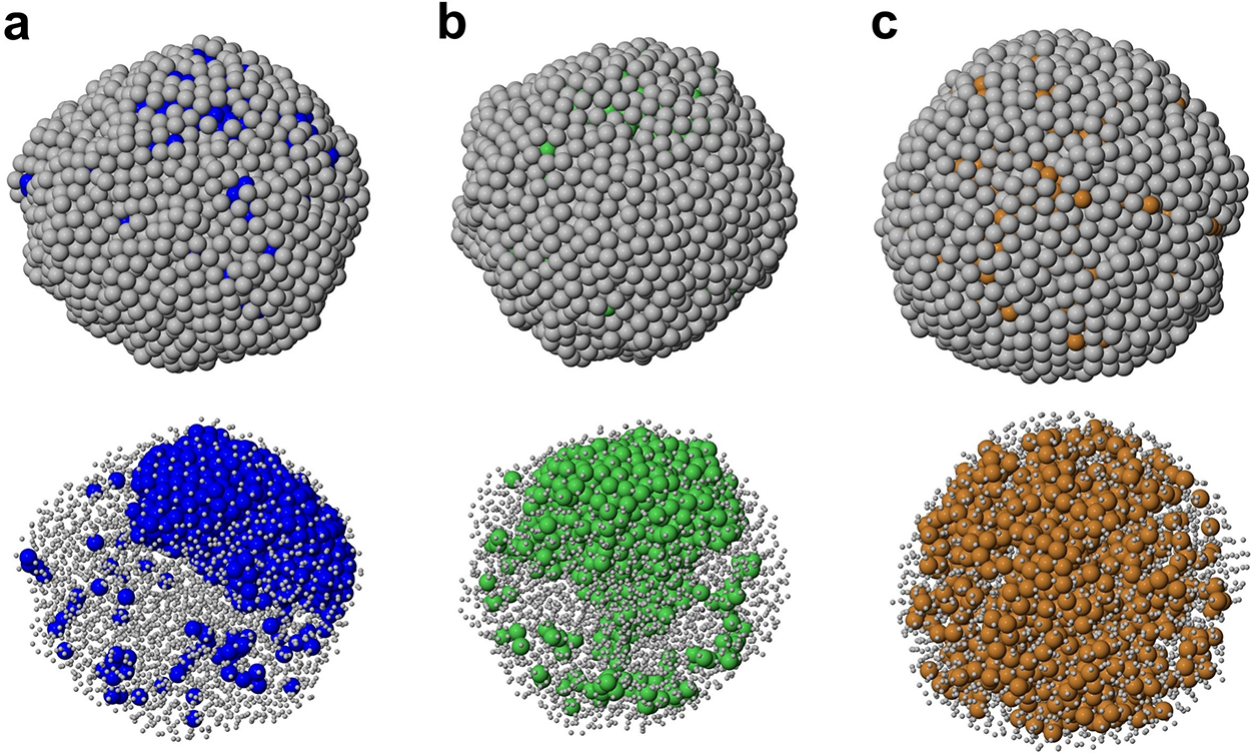
Representative snapshots of the structures taken at *T* = 1070 K. (a) AgCo, (b) AgNi, (c) AgCu. Ag atoms are colored in gray, whereas Co, Ni, and Cu atoms are colored in blue, green, and orange, respectively. In the bottom row, Ag atoms are shown as small spheres, whereas X atoms, which are mostly inside the nanoparticles, are shown by larger spheres. In this figure and in the following ones, the structures are shown after local minimization to eliminate the effects of vibrations. All nanoparticles are in the liquid state, without any evidence of arrays of crystalline planes. In AgCo and AgNi nanoparticles, there is already a quite clear phase separation between the elements, while in AgCu there is intermixing in the inner part of the nanoalloy.

Here we analyze the final outcomes of the freezing simulations for Ag_3000_X_1000_, comparing them with the initial configurations in the liquid. This size is chosen since it is representative of the general behavior that we find in the whole size range. The comparison of the final structures will allow one to identify the main differences between AgCo and AgNi, on one hand, and AgCu on the other. In Figure 1 we report snapshots at *T* = 1070 K, obtained in simulations at which the nanoparticles were thermalized at *T* = 1100 K and then cooled down with a rate of 1 K/ns. At this temperature all nanoparticles are in the liquid state. Even though the initial chemical ordering was completely random, the fast atomic mobility quickly produced notable changes. In all structures of Figure 1, the surface layer is almost completely made of Ag atoms, while X atoms are confined in the inner part of the nanoparticles. As reported in Table 1, the number *N*_surf_ of surface atoms of species X is between 10 and 30 over 1000. However, there are clear differences between AgCo/AgNi and AgCu, because in AgCo and AgNi the great majority of Co and Ni atoms are aggregated in a compact off-center core, while in AgCu, Cu atoms are almost uniformly dispersed in the inner part of the nanoparticle. The results of our simulations correspond well to the phase separation in the liquid part of the respective bulk phase diagrams for AgCo and AgNi^34,35^ and to the intermixing in the bulk liquid for AgCu.^36^

**Table 1:** Results for Ag_3000_X_1000_ (X = Co, Ni, Cu)^a^

*T* = 1070 K	*N*_s,X_	*N*_aggr,X_	*M*_X_	*R*_g,X_	*R*_g,X_/*R*_g_	*R*_g,X_/*R*_s,X_	*b*_X_	*n*_X_
AgCo	25 ± 3	44 ± 2	920 ± 10	11.5 ± 0.1	0.60 ± 0.01	1.22 ± 0.01	31 ± 3	8.5 ± 0.1
AgNi	11 ± 1	60 ± 3	900 ± 10	11.4 ± 0.1	0.60 ± 0.01	1.22 ± 0.01	26 ± 3	8.1 ± 0.1
AgCu	25 ± 2	74 ± 3	810 ± 20	17.0 ± 0.2	0.88 ± 0.01	1.84 ± 0.02	60 ± 8	4.3 ± 0.1

*T* = 400 K	*N*_s,X_	*N*_aggr,X_	*M*_X_	*R*_g,X_	*R*_g,X_/*R*_g_	*R*_g,X_/*R*_s,X_	*b*_X_	*n*_X_
AgCo	6 ± 1	8 ± 1	991 ± 1	11.1 ± 0.1	0.58 ± 0.01	1.15 ± 0.01	23 ± 3	10.2 ± 0.1
AgNi	5 ± 1	9 ± 1	992 ± 1	10.9 ± 0.1	0.57 ± 0.01	1.13 ± 0.01	13 ± 2	10.1 ± 0.1
AgCu	17 ± 2	67 ± 2	780 ± 40	14.8 ± 0.6	0.78 ± 0.03	1.62 ± 0.05	80 ± 11	6.1 ± 0.1
AgCu, 0.1 K/ns	11 ± 2	63 ± 3	820 ± 30	15.8 ± 0.4	0.83 ± 0.03	1.70 ± 0.03	96 ± 8	6.4 ± 0.1

a *N*_s,X_ is the number of X atoms in the surface layer, *R*_g,X_ is the gyration radius of the largest X aggregate (in Å); *b*_X_ is the asphericity of the largest X aggregates (in Å^2^). *M*_*X*_ and *R*_g,X_ (in Å) are the number of atoms in the largest X aggregate and its gyration radius; *R*_g_ is the gyration radius of the complete nanoparticle; *R*_s,X_ is the gyration radius of a spherical aggregate of 1000 X atoms; *n*_X_ is the average number of X nearest neighbors per X atom; *N*_aggr_ is the number of X aggregates (also isolated X atoms count as aggregates). All quantities are averaged over 10 independent simulations. The errors correspond to the standard deviations of the averages. The freezing rate from 1070 to 400 K is 1 K/ns if not otherwise specified.

Let us more deeply analyze nanoparticle shape and chemical ordering in the liquid state at *T* = 1070 K. We characterize the nanoparticle shape by the gyration radius *R*_g_ and by the asphericity *b*. Specifically, *R*_g_ is the gyration radius of the whole nanoparticle, which is calculated from the eigenvalues  of the gyration tensor as ; *b* is the asphericity of the nanoparticle, defined as , where  is the largest of the three eigenvalues. The asphericity *b* is always non-negative and zero only when the eigenvalues are all equal, i.e., when the aggregate is spherically symmetric. All data are taken after local minimization of the structures to eliminate the effects of thermal vibrations.

The average values of *R*_g_ are quite similar for the three systems, being in the range from 19 to 19.2 Å. Nanoparticle shapes are nearly spherical, since the differences between the principal radii in the gyration tensors are much smaller than *R*_g_, being in the range of 1–3 Å. However, the values of the asphericity show that AgCo and AgNi nanoparticles are slightly less spherical than AgCu ones. This is due to the accumulation of Co and Ni on one side of the nanoparticle, just below the surface, which causes a small bulge on the surface.

Let us now characterize the aggregation of X atoms in the inner part of the nanoparticle, by analyzing the distribution of the aggregates of species X. An aggregate is defined as a collection of atoms of a given species that can be connected to each other by a chain of nearest-neighbor bonds. Two X atoms are considered as nearest neighbors if their distance does not exceed the nearest-neighbor distance in their bulk crystal multiplied by 1.15, which gives 2.875 Å for Co, 2.864 Å for Ni, and 2.944 Å for Cu. In Table 1 we report the number *N*_aggr,X_ of aggregates of species X and characterize the largest of these aggregates by *M*_X_, *R*_g,X_, and *b*_X_, i.e., the number of atoms, the gyration radius, and the asphericity of the largest aggregate. In *N*_aggr,X_ also isolated X atoms (i.e., X atoms fully surrounded by Ag atoms) are counted as aggregates (of size 1).

From the data in Table 1 for *T* = 1070 K, it turns out that there are many aggregates (*N*_aggr,X_ in the range of 40–70), but there is always a dominant aggregate containing the great majority of X atoms, about 90% in AgCo and AgNi and about 80% in AgCu. The remaining X atoms are grouped into much smaller aggregates. The analysis of the shape of the largest aggregate by means of *R*_g,X_ and *b*_X_ better quantifies the differences between AgCo/AgNi and AgCu. In fact, *R*_g,Cu_ is quite close to *R*_g_, while *R*_g,Co_ and *R*_g,Ni_ are significantly smaller. *R*_g,Cu_ is also 80% larger than the gyration radius of a spherical aggregate of 1000 Cu atoms, while *R*_g,Co_ and *R*_g,Ni_ are only 20% larger than the radii of their respective spherical aggregates. These data confirm the ramified and noncompact character of the largest Cu aggregate, opposed to the compact ellipsoidal shape of the largest Co and Ni aggregates. This corresponds to a much larger degree of intermixing in AgCu. To further quantify intermixing, we calculate the average number *n*_X_ of X nearest neighbors per X atom. From the values in Table 1, we see that a Cu atom has on average about four Cu neighbors, while a Ni or a Co atom has about eight neighbors of the same species.

**Figure 2:**
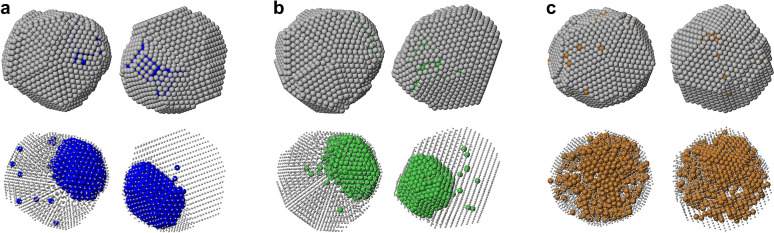
Representative snapshots of the nanoparticle structures at end of the simulations (*T* = 400 K). (a) AgCo, (b) AgNi, (c) AgCu. In all snapshots, for each system, we present the final structures of two independent simulations: for AgCo and AgNi there is an icosahedral (left) and an fcc (right) structure; for AgCu there is an icosahedral (left) and a decahedral (right) structure. Each structure is shown in two ways. In the top row, we show the nanoparticle surfaces, whereas in the bottom row Ag atoms are represented by small spheres to reveal the internal arrangement of the nanoparticles. All nanoparticles are solid, with clear evidence of well-defined crystal planes. In all systems, the surface layer is almost completely made of Ag atoms. We note that, in AgCo and AgNi, there is a neat phase separation, with Co and Ni atoms forming compact off-center cores, while for AgCu there is still some form of intermixing of the metals in the inner part.

Let us now analyze the final structures (see Figure 2) after they have cooled down to *T* = 400 K at a cooling rate of 1 K/ns. In all cases, the nanoparticles are solid, with well-defined atomic planes. For all systems, we find structures belonging to face-centered cubic (fcc), icosahedral, and decahedral motifs (see Table S1 of the Supporting Information, where we report the structural motifs obtained at the end of all freezing simulations). The shapes of the final aggregates are compact, with relatively small asphericity, whose values are close to those at *T* = 1070 K.

The main differences between the systems are found in the chemical ordering. In AgCo and AgNi, the cooling process causes an increase of phase separation. The largest Co and Ni aggregates increase their size to contain about 99% of the atoms (see Table 1). Correspondingly, the number of aggregates *N*_aggr,X_ is strongly decreased. The smaller aggregates are indeed a few Co or Ni atoms dispersed in the Ag matrix. The average number of bonds *n*_X_ increases to about 10, indicating that the largest aggregates are compact. Their asphericity *b*_X_ is slightly decreased compared to *T* = 1070 K, being therefore quite small. Their gyration radii *R*_g,Co_ and *R*_g,Ni_ are slightly decreased too, in spite of the fact that the number of atoms *M*_X_ is increased. In summary, in AgCo and AgNi, the cooling process leads to the full accumulation of all Co and Ni atoms in one aggregate whose shape tends to become more spherical.

The behavior of AgCu is quite different. *N*_aggr,Cu_ decreases only slightly, and the value of *M*_Cu_ is not significantly different from that at *T* = 1070 K. On the other hand, *R*_g,Cu_ decreases, and *n*_Cu_ increases, both in a significant way. However, *n*_Cu_ remains smaller than *n*_Co_ and *n*_Ni_ by four units. These data show an increase of phase separation in AgCu too, but this increase is not leading to the complete segregation of Cu into one large compact aggregate. The thin branches of the large aggregate formed in the liquid phase grow somewhat thicker (increase of *n*_Cu_), but in some cases, the thickening of an irregular branch may lead to its breaking into two pieces, with the formation of a new disconnected aggregate often containing several tens of atoms.

### Equilibrium Versus Kinetic Trapping in the Solid State

**Figure 3:**
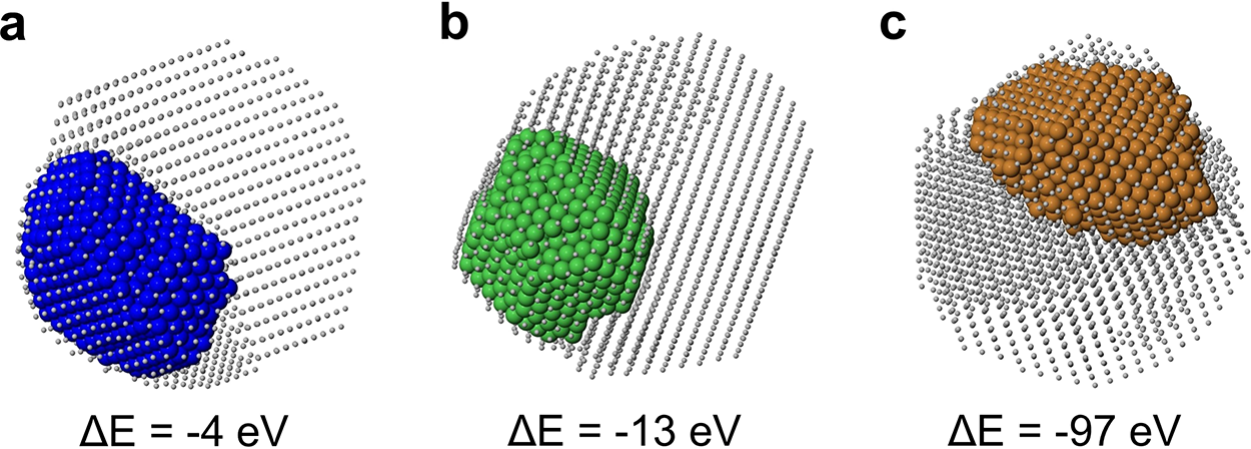
Optimal chemical ordering of the fcc and decahedral structures of Figure 2, with (a) AgCo, (b) AgNi, and (c) AgCu, as obtained by global optimization runs in which exchange moves only were employed. In all cases, the optimal structures present compact off-center X cores. The values of Δ*E* represent the energy gain after chemical ordering optimization.

Now we check whether our final configurations at *T* = 400 K are close to equilibrium. To this purpose, we take the lowest-energy structures found at *T* = 400 K for the different systems and optimize their chemical ordering. The results of the optimization are shown in Figure 3, in which we can see that there is complete phase separation with off-center cores in all systems, including AgCu. In the figure we also report the energy gain Δ*E* obtained by performing the optimization of chemical ordering. In AgCo and AgNi, Δ*E* is relatively modest, and this is due to the aggregation of all Co/Ni atoms and to some smoothing of the interface between Co/Ni and Ag. In AgCu, Δ*E* is much larger, because the ramified aggregates are eliminated in favor of a compact quasi-spherical aggregate of the same type as in AgCo and AgNi. These results clearly indicate that the structures obtained at *T* = 400 K are close to thermodynamic equilibrium for AgCo and AgNi, while in AgCu the structures are still far from equilibrium, since chemical ordering is kinetically trapped in a kind of intermixed state of very high energy.

The nonequilibrium character of the final configurations for AgCu at *T* = 400 K is further confirmed by the comparison with the results of ref (37), in which Monte Carlo simulations were made to determine the equilibrium chemical ordering in Cu-rich AgCu nanoparticles of sizes up to 2000 atoms. The results demonstrated that, in the liquid phase, there is intermixing between Ag and Cu in the inner part of the nanoparticles, however, with a mixing degree that was characterized as not completely random. These results agree well with our findings for AgCu at *T* = 1070 K. On the contrary, the equilibrium chemical ordering in the solid phase was found to be strongly phase-separated, which does not agree at all with the chemical ordering that we find after the phase had cooled down to *T* = 400 K.

In order to check whether equilibrium chemical ordering of AgCu can be obtained by a slower cooling process, we performed simulations at rate 0.1 K/ns, i.e., 10 times slower. The results are summarized in Table 1. The slower cooling rate allows some slightly better approach to the equilibrium state (increase of *n*_Cu_ and smaller energy gain Δ*E*, which goes from −97 to −72 eV), but the configuration at *T* = 400 K is still quite intermixed and very high in energy. These results indicate that full equilibration of AgCu chemical ordering is quite slow, well-beyond the time scale presently achievable by MD simulations.

Now it is interesting to check whether this difference in approaching equilibrium persists down to smaller sizes. To this end, we performed simulations at sizes *N* = 250, 500, 1000, and 2000, at the same composition 75 atom % of Ag and 25 atom % of X and with different cooling rates. In particular, for size 250 we performed the same analysis as at size 4000, which confirms that the equilibration of chemical ordering is better achieved in AgCo and AgNi than in AgCu down to very small sizes (see the Supporting Information, Figure S1).

### One-Step Versus Two-Step Solidification Processes

**Figure 4:**
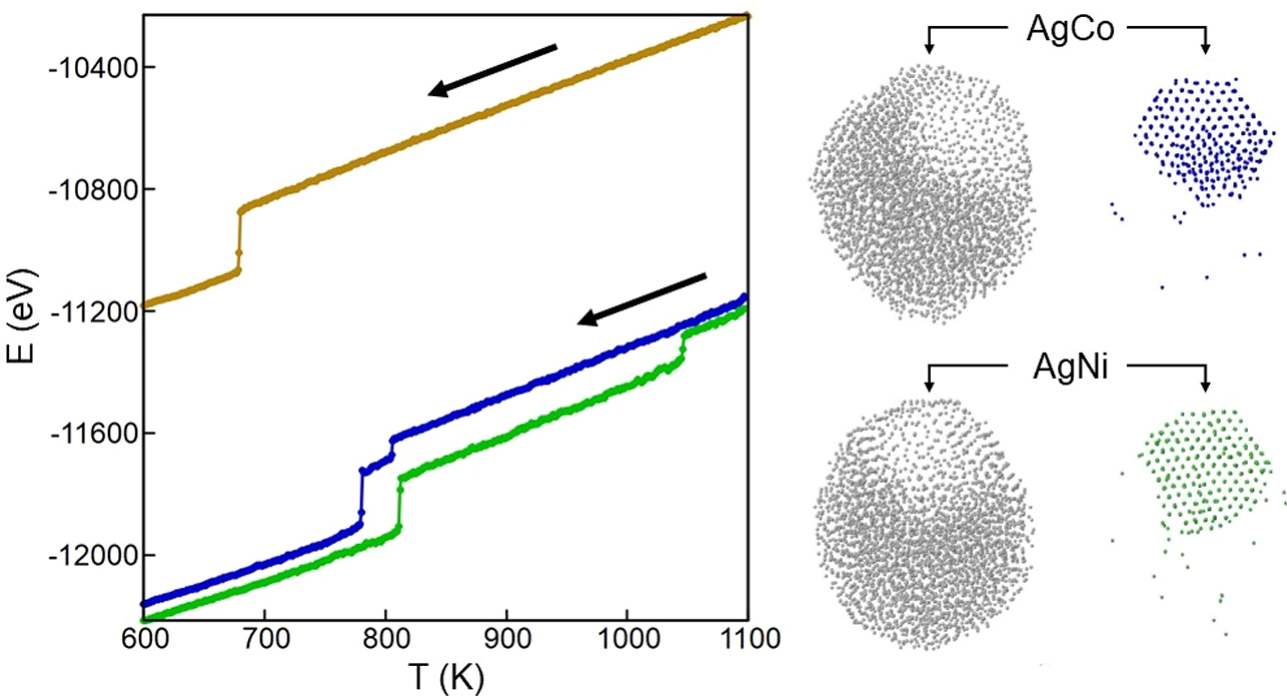
(left panel) Energy *E* (in eV) vs temperature *T* (in K): Ag_3000_Co_1000_ (blue), Ag_3000_Ni_1000_ (green), and Ag_3000_Cu_1000_ (orange). The arrows indicate that the temperature is decreasing in the simulations. The systems are cooled down in steps of 1 K every ns; *E*(*T*) is the average energy at temperature *T*. (right panel) Snapshots from the simulations of Ag_3000_Co_1000_ and Ag_3000_Ni_1000_ taken at *T* = 800 and *T* = 850 K, respectively. The atoms of the two species are shown separately. At these temperatures, the Ag part is still mostly liquid (compare with the solid Ag parts of Figure 2), while the Co and Ni parts are solid, with aggregates showing well-defined crystalline planes.

After comparing the initial structures in the liquid phase to the solid structures obtained at the end of the cooling process, we analyze the solidification pathways. In Figure 4 we report typical examples of the caloric curves (average energy *E* vs temperature *T*) obtained at a cooling rate of 1 K/ns. These curves show a further qualitative difference between AgCo/AgNi and AgCu. Starting from high temperature and going down in the direction of the arrows in Figure 4 one encounters jumps in the caloric curves. For AgCo and AgNi two well-separated jumps are visible, while in AgCu there is only one jump. In fact, in AgCo and AgNi, the minority element, which is also more cohesive, solidifies at higher temperature (first, high-temperature jump), so that there is a temperature range in which the Co and Ni parts are solid, while the Ag part is still liquid. This point will be better discussed in the following, in which we will show that the solid part is covered by a thin Ag crust. The second jump then corresponds to the solidification of Ag. In Figure 4 we also show snapshots of AgCo and AgNi nanoparticles taken in the temperature interval between the two jumps in the energy. The Co and Ni parts present well-defined crystalline planes confirming their passage to the solid state, while most of Ag is still liquid. On the contrary, in AgCu both elements solidify together.

The two-step solidification process of AgCo and AgNi is possible because of the clear phase separation of the two elements in the nanoparticle: there is a large aggregate containing almost all Co or Ni atoms and an Ag part in which very few atoms of the other species are dispersed. In AgCu the degree of phase separation is much milder. The Cu aggregates can be large, but they are not compact, with thin branches in which Cu atoms have several Ag neighbors, as we previously discussed. This peculiar structure does not allow a separate solidification.

**Figure 5:**
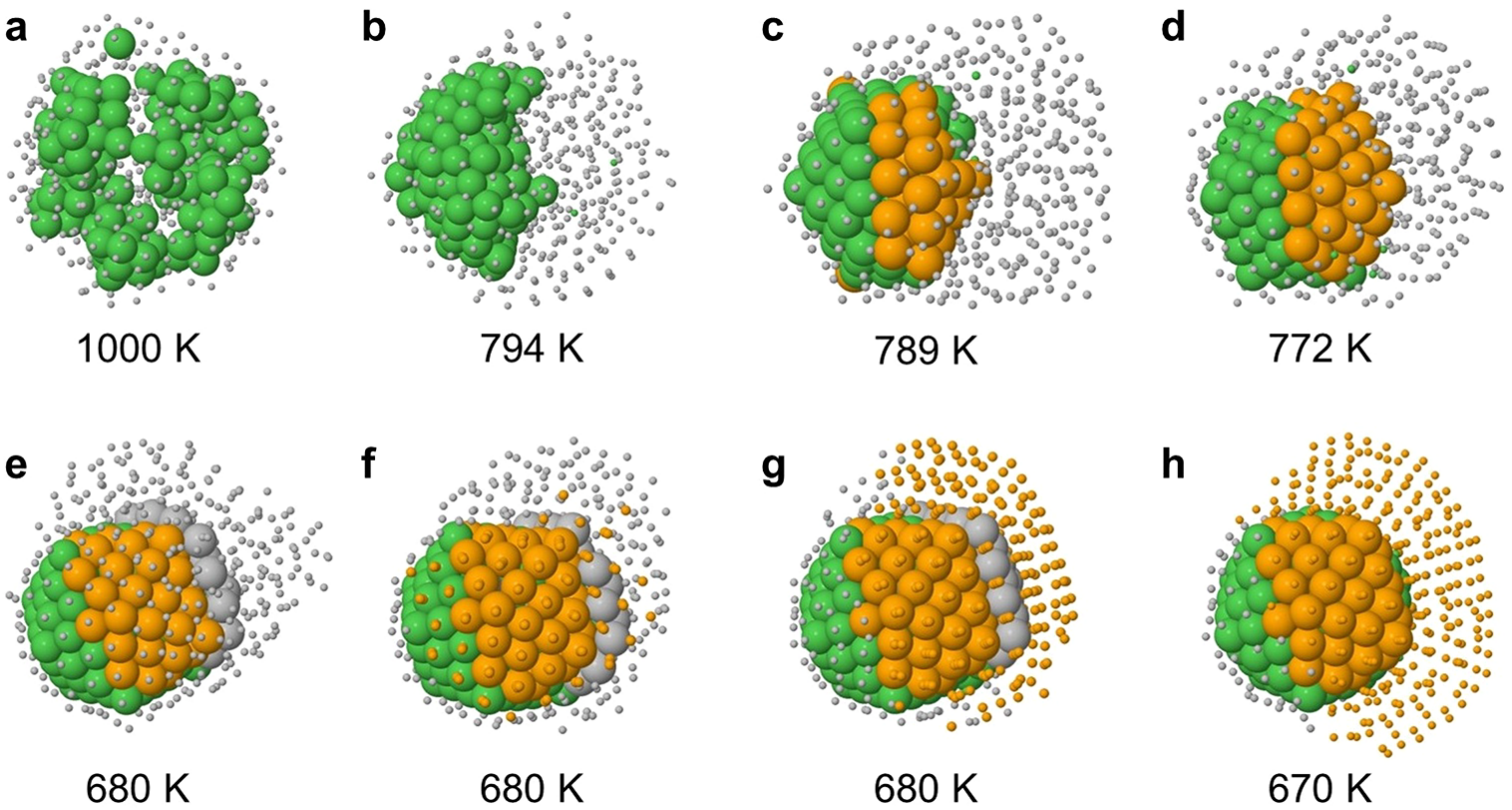
Representative snapshots from a freezing simulation of Ag_375_Ni_125_ with cooling rate of 1 K/ns. In all snapshots, Ni atoms are shown as green spheres. Ag atoms are represented with different sizes (spheres and dots) and colors (gray and orange) depending on their state: gray dots correspond to Ag atoms in the liquid state; orange spheres correspond to Ag atoms belonging to the Mackay icosahedral solid nucleus of 147 atoms; gray spheres represent Ag atoms forming an icosahedral layer (mostly anti-Mackay) on top of the icosahedral nucleus; orange dots represent other Ag atoms in the solid state.

Let us take a closer look at the two-step solidification process. To this end, in Figure 5, we report snapshots from a representative simulation of the freezing of Ag_375_Ni_125_ with a cooling rate of 1 K/ns. We choose this size because it is sufficiently small to follow the process atom by atom.

The first step of the solidification takes place between snapshots (b) and (c) and consists in the formation of a solid nucleus containing all 125 Ni atoms (Figure 5b). This nucleus is off-center and rather compact, but it is far from being spherical. In fact, its surface is somewhat stretched to place a large proportion of Ni atoms in subsurface positions, which are energetically favorable for small atoms in a matrix of larger atoms,^38^ because in these positions a better release of atomic-level stress^39^ can be achieved. This nucleus is enlarged by some Ag atoms (Figure 5c), which progressively solidify and complete the 147-atom Mackay icosahedron (Figure 5d). This complete icosahedral nucleus is shown from different views in the Supporting Information, Figure S2. The icosahedral nucleus persists, as it is in a wide temperature range, from about 772 down to 680 K. At this temperature the solidification of the remaining Ag atoms takes place (Figure 5e–g). The 147-atom nucleus begins to be covered partially by a Ag solid monolayer, which is initially placed on anti-Mackay stacking (corresponding to placement on hexagonal close-packed (hcp) sites of the (111)-like facets of the icosahedron^40^). This triggers the solidification of all Ag atoms (Figure 5g). Finally the Ag part reverts to the Mackay stacking (Figure 5h), with the exception of the Ag atoms covering the Ni nucleus on the left side of Figure 5h, which keep the anti-Mackay stacking.

These results show that the two-step solidification process is not simply the solidification of the Ni part followed by that of the Ag part at lower temperatures. Instead, the solid nucleus of the first step can contain also Ag atoms, which contribute to complete a geometric *magic* structure.

### Size Dependence of the Solidification Temperature

Here we address the problem of the size dependence of the solidification temperature, by analyzing the behavior of *T*_sol_, which is the temperature at which the whole nanoparticle becomes solid. Previous simulations on the solidification of single-component metallic nanoparticles were not able to show any significant size dependence of *T*_sol_, neither in microcanonical nor in canonical freezing simulations.^10^ This finding was attributed to solidification starting in the inner part of the nanoparticles by homogeneous nucleation processes that present size-independent free-energy barriers for the creation of stable solid nuclei.^10^ At variance with these results, our simulations below show a clear dependence of *T*_sol_ on size in our binary systems, with qualitative and quantitative differences between AgCo/AgNi and AgCu.

**Figure 6:**
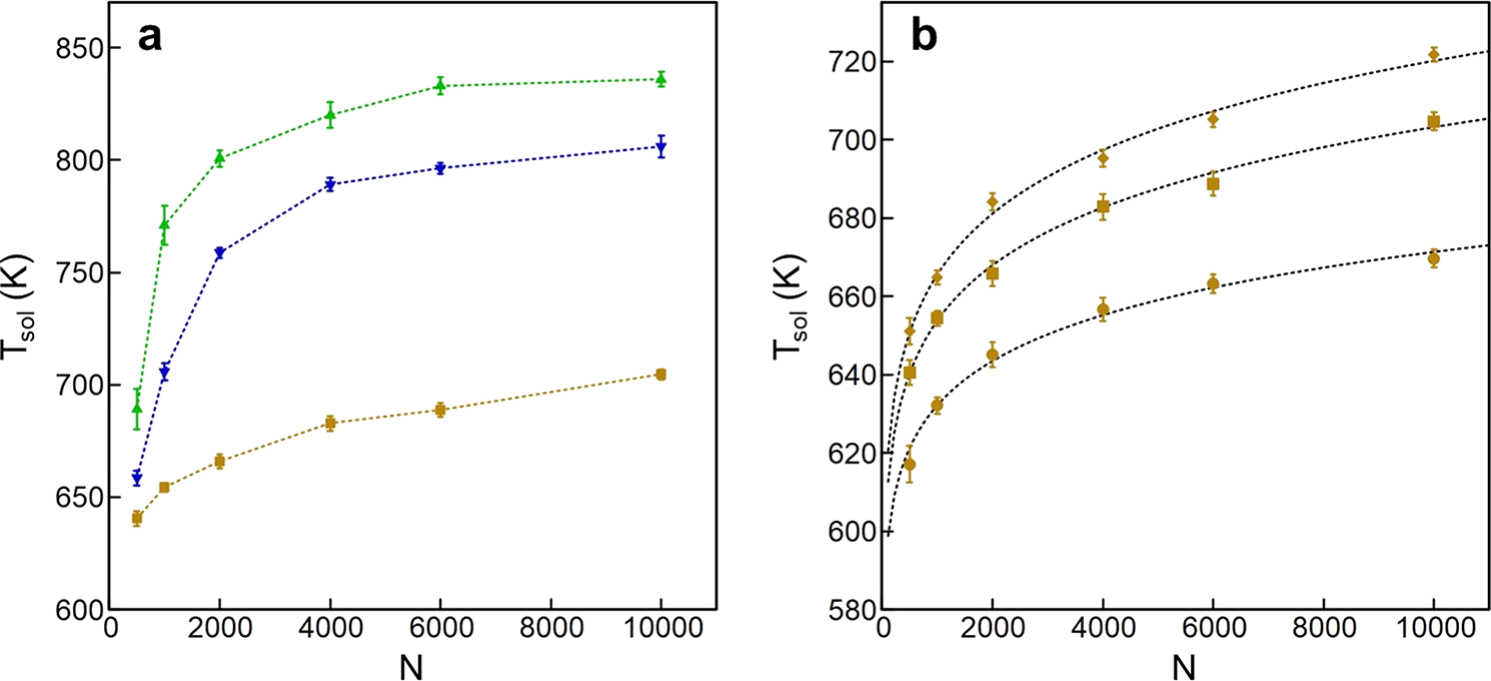
(a) Size dependence of the solidification temperature *T*_sol_ (in K) of AgCo (blue downward triangles), AgNi (upward green triangles), and AgCu (orange squares) nanoparticles. All data are obtained with a cooling rate of 1 K/ns, averaging over 10 independent simulations. The dotted lines are only guides to the eye. Error bars correspond to one standard deviation on the average *T*_sol_. (b) Size dependence of the solidification temperature (in K) of AgCu nanoparticles at the cooling rates of 10 K/ns (circles), 1 K/ns (squares), and 0.1 K/ns (diamonds). The lines correspond to the best fits of eq 7. The values of the fits for α and γ in eq 7 are given in Table S2.

In order to investigate the size dependence of solidification in our binary systems, we performed 10 independent simulations per system and temperature, in the size range *N* = 500–10000 at a cooling rate of 1 K/ns. The results are reported in Figure 6a, where we plot the temperatures *T*_sol_ at which the entire nanoparticle becomes solid. Details on the calculation of the solidification temperatures are given in the Supporting Information (Figure S3 and comments). It turns out that the solidification temperature does depend on *N* for all systems but more strongly in AgCo and AgNi than in AgCu—from *N* = 500 to 10000 there is an increase of 150/160 K in AgCo/AgNi and of 70 K in AgCu. In addition to these quantitative differences, size-dependent behavior also appears qualitatively different. In AgCo/AgNi there is a sharp rise of *T*_sol_ from *N* = 500 to 2000, followed by some sort of flattening that might allude to saturation. In AgCu, the slow rise shows no sign of saturation. We now demonstrate that these results stem from different solidification mechanisms of AgCo/AgNi and AgCu, indicating that the crucial point is the presence or absence of phase separation prior to solidification.

Let us analyze AgCu first. In Figure 6b we report the data of *T*_sol_ for three cooling rates, namely, 10, 1, and 0.1 K/ns. For all rates we find that *T*_sol_ significantly depends on *N*, with the same type of slow rise. Here we rationalize this size dependence using classical nucleation theory and a low-temperature expansion of the free-energy barrier for the nucleation of the solid phase.

Following ref (3), the nucleation rate *r*_nuc_ in the cooling droplet can be expressed as
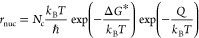
1where *N*_c_ is the number of nucleation centers in the droplet, ΔG* is the free energy barrier for the formation of a stable solid nucleus, and *Q* is the activation free energy for transporting an atom across the solid–liquid interface,^41^ which is typically of the order of the barriers for atomic motion in the droplet.^4^ Given the homogeneous character of the AgCu nanoparticles, we assume that *N*_c_ is proportional to the size of the droplet, since nucleation can occur at any place. This means that *N*_c_ = *cN*, where *c* is a constant. This assumption and its generalizations are discussed in the Supporting Information.

Following the observations in ref (10), we expect that solidification occurs somewhat above the temperature *T*_inst_ at which the liquid phase becomes thermodynamically unstable. At *T*_inst_, ΔG* vanishes.^4,42^ Here we assume that, for *T* ≥ *T*_inst_, ΔG* can be approximated by the following first-order expression
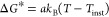
2where *a* is a dimensionless constant. From eqs 1 and 2 one finds
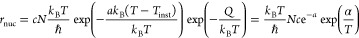
3where α = *aT*_inst_ – *Q*/*k*_B_. If we neglect the temperature dependence in the slowly varying factor *k*_B_*T*/*ℏ* with respect to that of the rapidly varying exponential, we obtain the expression
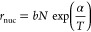
4where *b* is a constant. The stable nucleus forms at the temperature where the inverse of *r*_nuc_ equals the typical observation time τ_obs_, which is of the order of the inverse cooling rate. Therefore, *T*_sol_ is determined by
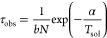
5which gives
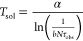
6which is of the form
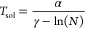
7with γ = – ln(*bτ*_obs_).

eq 7 is used to fit the simulation data of Figure 6b. It turns out that eq 7 very well describes the size dependence of the solidification temperature, with very good quantitative agreement for all cooling rates. The parameters of the fit are reported in Table S2 in the Supporting Information, together with a further analysis of this model.

When applied to AgCo and AgNi, eq 7 fails to provide an acceptable fit of the simulation data, even qualitatively (see the Supporting Information, Figures S5 and S6, and related discussion). This can be understood by considering the different solidification mechanism, which has been discussed above in relation to Figure 5. There, we have shown that the Ag-rich part solidifies at a specific defect, which is the already solid Ni-rich part. This is a kind of heterogeneous nucleation mechanism in which the number of nucleation centers *N*_c_ is not likely to scale with *N* in a simple way.

## Conclusions

Solidification pathways in AgCo, AgNi, and AgCu differ in several aspects. AgCo and AgNi exhibit a net phase separation between the two elements already in the liquid phase, with the Co- or Ni-rich part placed in off-center positions and covered on one side by a Ag monolayer. This triggers a solidification process in which the nanoparticle can easily reach low-temperature configurations whose chemical ordering is very close to the lowest energy (optimal) one, which tends to represent the equilibrium configuration at sufficiently low temperatures.^37^ For this reason, we expect that, even for much slower cooling rates, such as those of many experiments, the qualitative behavior upon freezing should be of the same type. On the contrary, in AgCu, we were not able to detect any clear phase separation in the inner part of the nanoparticle—note that the nanoparticle surface is strongly enriched in Ag for all systems. After solidification, a notable degree of intermixing of Ag and Cu was present, a situation that did not change upon cooling at the solid state down to room temperature. For this reason, the AgCu solid nanoparticles were always quite far from the equilibrium chemical ordering at room temperature. This behavior of AgCu was very weakly sensitive to changes in the cooling rate by 2 orders of magnitude, from 10 to 0.1 K/ns, pointing to a very long lifetime of the metastable intermixed configurations in the room-temperature range.

The long lifetime of metastable AgCu intermixed nanoparticles in our simulations can explain a series of experimental observations.^43−47^ In these experiments, intermixed AgCu nanoalloy catalysts were produced, with typical nanoparticle sizes corresponding to those of our simulated nanoparticles. The nanoparticles were produced by procedures in which the initial part takes place at high temperature, and then the nanoparticles were shown to be stable at room temperature for long times. Evidence of AgCu nanoparticles with pure Ag shell, intermixed internal part, and good crystalline order was also given,^44,45^ in perfect agreement with our simulations.

A further difference between AgCo/AgNi and AgCu is the occurrence of a two-step solidification pathway, which has been observed in the simulations of AgCo and AgNi but not in those of AgCu. Two-step solidification is triggered by the phase separation in the liquid, which allows the Co- or Ni-rich part to solidify first. However, a closer inspection of the simulations of Ag_375_Ni_125_ shows that the solid part formed in the first step is not simply a Ni aggregate but a Ni aggregate completed by Ag atoms to form an especially stable structure.

The generalization of our results to other systems is far from trivial. Reference to bulk equilibrium phase diagrams can be a useful guide but with several complications arising from the finite size of the systems and the presence of kinetic effects in the freezing process. Anyway, one may expect that systems showing a clear phase separation in the liquid state (e.g., AuRh) present a freezing behavior similar to that of AgNi and AgCo. On the other hand, systems in which there is a range of temperatures in which solid solutions form for all compositions, while the miscibility gap is only at lower temperatures (such as AuPt and CuNi^48^), should solidify in intermixed configurations, which may persist down to low temperatures where they become out of equilibrium.

Our simulations have shown that the final solidification temperature *T*_sol_ (at which the whole droplet becomes solid) depends on size for all systems shown. However, the size dependence is qualitatively different and quantitatively stronger in AgCo and AgNi compared to AgCu. This difference has been attributed to the occurrence of two solidification mechanisms, i.e., heterogeneous nucleation of Ag at the interface between the two phase-separated parts for AgCo/AgNi and homogeneous nucleation for AgCu, in which our analytical model has shown that a logarithmic size dependence of the solidification temperature holds.

## Methods

### Model

Interactions between nanoparticles were modeled by an atomistic potential derived from the second moment approximation to the tight-binding model,^49^ which can be found, for example, in ref (50). The parameters of the potential are taken from refs (51), (52), and (53) for AgCu, AgCo, and AgNi, respectively. This interaction potential has been used in several works to model the structures of AgCu, AgCo, and AgNi nanoalloys, obtaining a good agreement with experimental results and density functional theory calculations.^28,30,40,54,55^

### Simulation Methods

Freezing simulations were performed by classical Molecular Dynamics (MD) using our own codes, in which Newton’s equations of motion are solved by the Velocity Verlet algorithm with a time step of 5 fs, which can be safely used to simulate metallic systems up to high temperatures in the liquid state.^56^ The simulations of nanoparticles for sizes up to 2000 atoms were performed by the CPU version of the code, while those for larger sizes were computed on the GPU version (developed with the NVIDIA CUDA library). In the GPU version, particular attention has been paid to the method for the neighbor search. The method used for this step is the one described in ref (57), which is based on Verlet lists constructed from a grid. Since clusters evolve in the whole space, the indexes of the grid are associated with a set of cells as
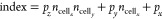
8with , , and  the number of grid cells in *x*, *y*, and *z* directions, respectively, and
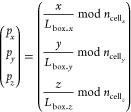
9with *L*_box.*i*_ the length of the simulation box in the *i*th direction. In this way all the atoms of a cell are grouped in the same index. In general, for small sizes up to 500 atoms the CPU version is faster, while between 500 and 1000 atoms both versions run at comparable speed. For sizes above 1000 atoms, the GPU code becomes progressively much more efficient than the CPU code.

The initial configurations of the simulations are truncated octahedra in the solid state, with random chemical ordering.^50^ These structures are equilibrated for at least 10 ns at high temperatures (*T* in the range of 900–1100 K, depending on system and on nanoparticle size). At these temperatures, the nanoparticles rapidly lose memory of the initial configuration, reaching the liquid state and equilibrating their chemical ordering, due to the high mobility of atoms in the liquid state. For AgCo and AgNi, we ran some additional simulations using the same procedure but changed the initial temperature to 1300 and 1400 K instead of 1100 K. This change did not produce any significant difference in freezing behavior. In most simulations, the nanoparticles were cooled down with a rate of 1 K/ns, which is a realistic cooling rate for a metal nanoparticle in a relatively dense inert gas.^58^ Additional simulations were performed by cooling down faster (at 10 K/ns) and slower (0.1 K/ns), in order to check the dependence of the results on the cooling rate. In all cases, temperature is lowered down from the initial one by small steps of 1 K, so that the different cooling rates were obtained by changing the frequency at which the temperature is lowered. This was done to avoid large sudden temperature jumps that would be unrealistic. We note that our cooling rates are much slower than in other recent MD simulations of nanoalloys.^20,23^ The simulations were stopped well-below the solidification temperature, i.e., at *T* = 400 K or lower. For each system, size, and cooling rate, 10 independent simulations were run.

For the smallest nanoparticle size (*N* = 250), global optimization searches were performed by the basin hopping algorithm^59^ by using the search strategies explained in ref (60), which are based on the use of both shape-changing and atomic exchange moves. In all cases, at least five independent searches of 10^5^ steps each were performed. At size *N* = 4000, chemical ordering optimization searches at fixed geometric shape were performed, with the employment of exchange moves only, as in ref (54). Three independent simulations of 10^5^ were performed for each system.

## Supporting Information Available

Dependence of the final structures on nanoparticle size and cooling rate; kinetic trapping down to small sizes; solid nucleus in AgNi; examples of caloric curves; alternative models for *T*_sol_; parameters of the fits in Figure 6b of the main text; comparison of the models with the simulation data for AgCu; solidification temperatures of AgCo and AgNi (PDF)
