# Supplementary material for: Two-Steps Versus One-Step Solidification Pathways of Binary Metallic Nanodroplets
Source: ACS Nano. 2022 Dec 20;17(1):587–96. doi: 10.1021/acsnano.2c09741 (PMC9836354; doi:10.1021/acsnano.2c09741)
Supplement: Supplementary file 1 — nn2c09741_si_001.pdf [file nn2c09741_si_001.pdf]

Supporting Information for Two-Steps Versus One-Step Solidification Pathways of Binary Metallic Nanodroplets

Diana Nelli,[*,†] El Yakout El Koraychy,[†] Manuella Cerbelaud,[*,‡] Benoit Crespin,[¶] Arnaud Videcoq,[‡] Alberto Giacomello,[§] and Riccardo Ferrando[*,†]

†*Dipartimento di Fisica, Università di Genova, Via Dodecaneso 33, 16146 Genova, Italia*

‡*Université de Limoges, CNRS, IRCER, UMR 7315, F-87000 Limoges, France*

¶*Université de Limoges, CNRS, XLIM/ASALI, F-87000 Limoges, France*

§*Dipartimento di Ingegneria Meccanica e Aerospaziale, Sapienza Università di Roma, via Eudossiana 18, 00184 Roma, Italia*

E-mail: diana.nelli@edu.unige.it; manuella.cerbelaud@unilim.fr; ferrando@fisica.unige.it

Dependence of the final structures on nanoparticle size and cooling rate

Here we consider nanoparticles of fixed composition (75 at% Ag) and different sizes, N=250, 500, 1000, 2000, 4000, in order to study the dependence of the final structures on size and cooling rate. The results are reported in Table S1.

The main trend arising from these results is the disappearance of fcc and Dh structures with decreasing size, to the advantage of Ih structures, and, at the smallest size, of some polyicosahedra (polyIh). The polyIh structures are made of smaller icosahedra sharing some atoms. The predominance of Ih structures is common to all systems, and it is already evident

Table S 1: Final geometric structures for different sizes and cooling rates. The nanoparticle structures are classified as face centered cubic (fcc), decahedral (Dh), icosahedral (Ih) and polyicosahedral (polyIh). In all cases 10 independent simulations are run. All structures are defective, for example fcc structures very often present twin planes and/or hcp parts. Some decahedral structures are indeed bi- or tri-decahedra.[1]

Size	System	Rate (K/ns)	fcc	Dh	Ih	polyIh
250	AgCo	1	0	0	7	3
250	AgNi	1	0	0	0	10
250	AgCu	0.1	0	0	10	0
250	AgCu	1	0	0	9	1
250	AgCu	10	0	0	10	0
500	AgCo	1	0	0	10	0
500	AgCo	10	1	3	6	0
500	AgNi	1	0	0	9	1
500	AgNi	10	0	0	6	4
500	AgCu	1	0	0	9	0
500	AgCu	10	2	0	8	0
1000	AgCo	1	2	1	7	0
1000	AgCo	10	3	3	4	0
1000	AgNi	1	0	1	9	0
1000	AgNi	10	1	1	8	0
1000	AgCu	1	2	0	8	0
1000	AgCu	10	1	1	8	0
2000	AgCo	1	2	4	4	0
2000	AgCo	10	2	3	5	0
2000	AgNi	1	4	0	6	0
2000	AgNi	10	1	1	8	0
2000	AgCu	1	2	0	8	0
2000	AgCu	10	1	1	8	0
4000	AgCo	1	5	3	2	0
4000	AgNi	1	5	1	4	0
4000	AgCu	0.1	4	2	4	0
4000	AgCu	1	4	3	3	0

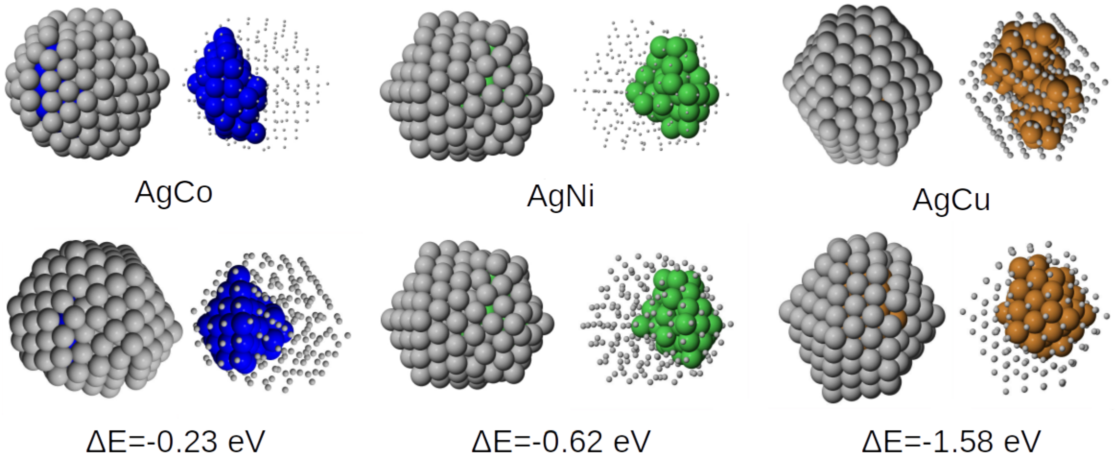

Figure S 1: Lowest energy structures found during the freezing simulations (top row) and structures with optimized chemical ordering after basin hopping searches with exchange moves (bottom row) for $Ag_{187}X_{63}$ nanoparticles (size 250). The energy gain ΔE after optimization is reported for each case.

for size $N = 2000$. For this size, the icosahedron is not the most favorable structure for the elemental nanoparticles of the metals considered here.[2] However, we note that in these binary nanoparticles, the icosahedron may become energetically favourable because the smaller Co, Ni and Cu atoms may help in releasing the strong compression in the central part of the structure. The cause of the abundance of icosahedra may be also related to kinetic trapping effects.

Kinetic trapping down to small sizes

Kinetic trapping effects are likely to be present even at the smallest size $N = 250$. This can be understood by comparing the lowest energy structures found in the cooling simulations with the optimized chemical ordering found by basin hopping searches, as for size 4000 (see main text). To this end, for all freezing simulations, we sample 20 structures at every temperature (remember that we lower the temperature by steps of 1 K), locally minimize them and select the lowest energy one. These lowest energy structures found in the freezing simulations are shown in the top row of Fig. S1. Then we take these nanoparticles and

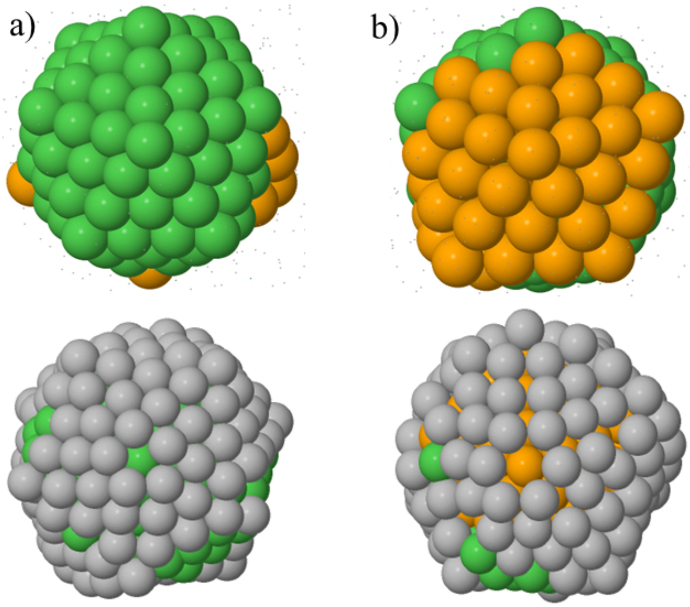

Figure S 2: Snapshot of Fig. 5(d) in different views and representations. (a): Top row, the icosahedral nucleus shown from the subsurface side; bottom row, the same nucleus covered by an Ag monolayer. (b): Top row, the icosahedral nucleus shown from side pointing to the internal part of the nanoparticle; bottom row, the same nucleus covered by an Ag monolayer, which is the embryo of the anti-Mackay layer. Colours and symbols as in Fig. 5 of the main text.

optimize chemical ordering by running 10^4 exchange moves (i.e. swaps of the positions of atomic pairs of different species) using the algorithm explained in Ref.[3] For all systems there is an energy gain (see the values of ΔE in Fig. S1) indicating that the chemical ordering obtained in the freezing simulations is not optimal. This is especially true for AgCu, in which the energy gain is by far the largest. Therefore, also for size 250, kinetic trapping is stronger in AgCu. To verify whether kinetic trapping persists on longer times scales, we performed 10 simulations of AgCu at the slower cooling rate of 0.1 K/ns, finding a somewhat smaller energy gain after optimization of chemical ordering ($\Delta E = -1.18$ eV), which is however larger than the ΔE values of AgCo and AgNi found at the faster cooling rate.

Solid nucleus in AgNi

In Fig. S2 we show the complete icosahedral nucleus which starts the solidification process in a freezing simulation of $Ag_{375}Ni_{125}$ with cooling rate 1 K/ns. The nucleus is the same as

in Fig. 5(d) of the main text.

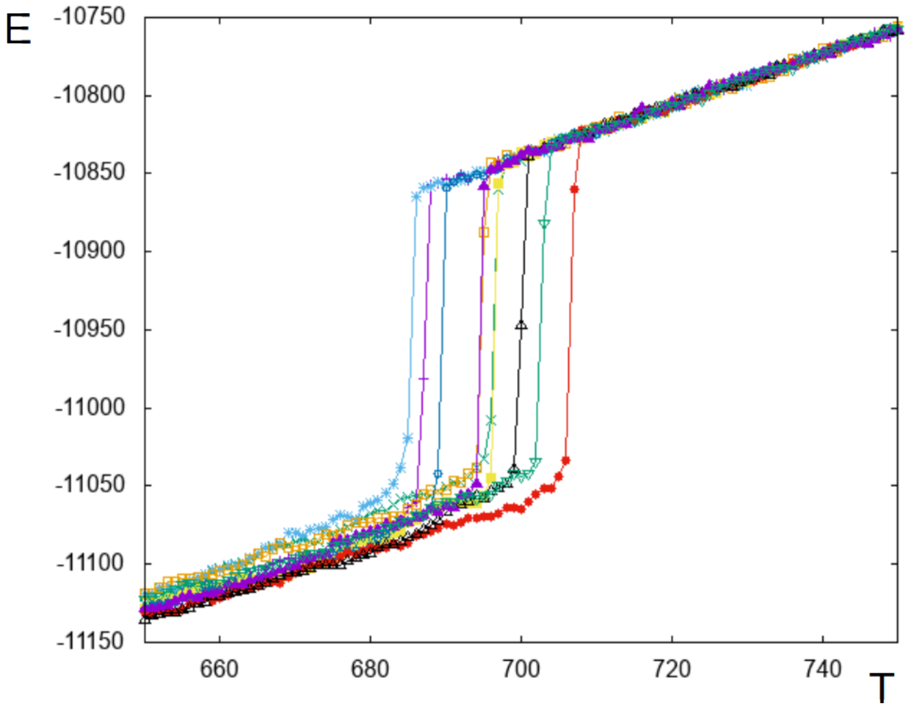

Figure S 3: Caloric curves of the 10 independent simulations of $Ag_{3000}Cu_{1000}$ with a cooling rate of 0.1 K/ns. The total average energy E (in eV) of the nanoparticle is reported as a function of the temperature T (in K).

Examples of caloric curves

In Figure S3 we report the caloric curves obtained in the 10 independent simulations of $Ag_{3000}Cu_{1000}$ with a cooling rate of 0.1 K/ns. The simulations started at $T = 1100$ K and were continued down to 400 K, but in the figure we show only the temperature range in which the jumps occur. In each simulation, the jump is quite sharp, but it changes its position from simulation to simulation.

Jumps are generally sharp for all cooling rates, with the exception of the solidification of small Ni-rich parts in which the solidifying part may oscillate between liquid and solid states in temperature intervals of the order of 10-15 K.

The solidification temperatures reported in the main text (Fig. 6(a,b)) have been cal-

culated as averages on 10 simulations. The error bars on these averages correspond to one standard deviation.

Alternative models for T_{sol}

We start from Eq. (1) of the main text,[4] which is repeated here

$$r_{nuc} = N_c \frac{k_B T}{\hbar} \exp\left(-\frac{\Delta G^*}{k_B T}\right) \exp\left(-\frac{Q}{k_B T}\right). \tag{S1}$$

If we assume that in general $N_c = cf(N)$, where f is a generic function, the expression for T_{sol} becomes

$$T_{sol} = \frac{\alpha}{\gamma - \ln(f(N))}. \tag{S2}$$

If $f(N)$ has a power-law behaviour, $f(N) = cN^\delta$, the functional form of T_{sol} becomes

$$T_{sol} = \frac{\alpha'}{\gamma' - \ln(N)}. \tag{S3}$$

where $\alpha' = \alpha/\delta$ and $\gamma' = \gamma/\delta$, so that this type of functional dependence of T_{sol} does not discriminate for example between droplets in which the density of nucleation centers scales as the volume and droplets in which nucleation centers scale proportionally to the surface area ($\sim N^{2/3}$).

An alternative approximation uses for T_{sol} the same functional of dependence as in the Pawlow's law[5] for equilibrium melting, i.e.

$$T_{sol} = \eta - \frac{\xi}{N^{1/3}}. \tag{S4}$$

Sheng et al.[6] used this kind of size dependence also for solidification, which is a kinetic phenomenon, in the case of heterogeneous nucleation of single-component nanoparticles. The justification of the use of this formula for solidification relies on several assumptions:

- Classical nucleation theory[4] is used for estimating the formation rate of a critical solid nucleus.

- In the expression of the nucleation rate (Eq. (S1)) the only size dependence is assumed to derive from the term corresponding to the free-energy difference per unit volume at equilibrium between liquid and solid phases, while N_c is assumed not to depend on N. The size dependence of the free energy difference is due to the interface free energy between the droplet and its environment.

- The free energy difference is evaluated by a first order expansion down from high temperatures. i.e. in $(T_{melt} - T)$, where T_{melt} is the bulk melting temperature of the metal.

Sheng et al. showed that this expression can nicely explain the size dependence of solidification for In, Sn, Bi, Cd, Pb nanoparticles embedded in an aluminum matrix.

Parameters of the fits of Figure 6(b) of the main text

The parameters α and γ of Eq. (7) of the main text i.e.

$$T_{sol} = \alpha/(\gamma - \ln(N)) \tag{S5}$$

used to fit the data of Fig. 6(b) of the main text are given in Table S2. For each cooling rate, we fit both parameters independently.

We note that, as derived in the main text, $\alpha = aT_{inst} - Q$, which should not depend on the transition rate. From the results in Table S2, it turns out that the α values for the cooling rates of 0.1 and 1 K/ns are quite close, since their difference is compatible with zero within 1.1 standard deviations. On the contrary, the α value for the cooling rate of 10 K/ns is significantly larger, thus being not compatible with the other values. This indicates that the linear expression of ΔG in terms of $(T - T_{inst})$ is able to to capture the main characteristics

Table S 2: Parameters of Eq. (7) in the main text (i.e. Eq. (S5)), corresponding to the fits represented by full lines in Fig. S4. Cooling rate is in K/ns and α is in K. The errors correspond to one standard deviation. The p-values refer to the χ-square test.

Cooling Rate	$\alpha \times 10^{-4}$	γ	p-value
0.1	2.02 ± 0.09	37.3 ± 1.2	0.28
1	2.14 ± 0.07	39.7 ± 1.0	0.77
10	2.49 ± 0.12	46.4 ± 1.8	0.72

Table S 3: Parameters of Eq. (S4) corresponding to the fits represented by dash-dotted lines in Fig. S4. Cooling rate is in K/ns, η and ξ are in K. The errors correspond to one standard deviation. The p-values refer to the χ-square test.

Cooling Rate	η	ξ	p-value
0.1	758 ± 7	920 ± 90	$2.4\ 10^{-5}$
1	737 ± 7	810 ± 80	$3.3\ 10^{-3}$
10	700 ± 2	683 ± 18	0.96

of the size dependence of T_{sol}, but it is probably much too simple for a full quantitative description of the dependence of T_{sol} on both size and cooling rate.

Comparison of the models with the simulation data for AgCu

In Figure S4 we compare the best fits of Eq. (S5) (solid lines) and of Eq. (S4) (dash-dotted lines) with the simulation data for AgCu (symbols). We already verified that Eq. (S5) very nicely fits the data for all cooling rates, with quantitative agreement. On the contrary, Eq. (S4) fits well only the data for the cooling rate of 10 K/ns, while the fits for 1 K/ns and 0.1 K/ns are poor from the quantitative point of view (see the p-values of the χ-square test in Table S3).

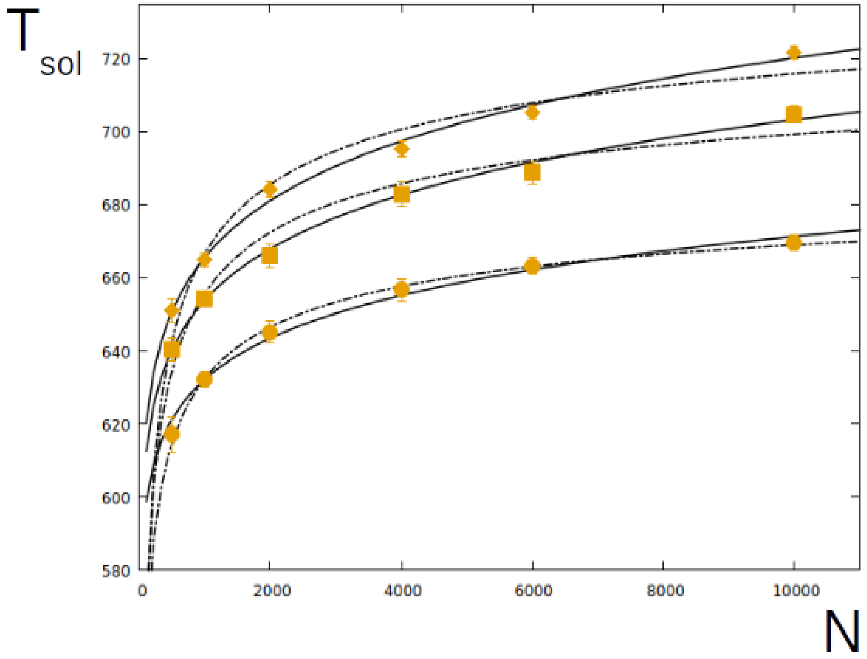

Figure S 4: Comparison of Eq. (S5) (solid lines) and of Eq. (S4) (dash-dotted lines) with the simulation data for the solidification temperature T_{sol} for cooling rates of 10 (circles), 1 (squares) and 0.1 (diamonds) K/ns. T_{sol} is in K. The error bars on the symbols correspond to one standard deviation of the average T_{sol} over 10 independent simulations.

Solidification temperatures of AgCo and AgNi

In Figures S5 and S6 we report the solidification temperatures for the Ag-rich parts and the Co/Ni rich parts, respectively. The simulation data are compared to the best fits of Eq. (S5) (solid lines) and of Eq. (S4) (dash-dotted lines). In all cases, no quantitative agreement is obtained, but Eq. (S4) in much better qualitative agreement with the simulation data than Eq. (S5).

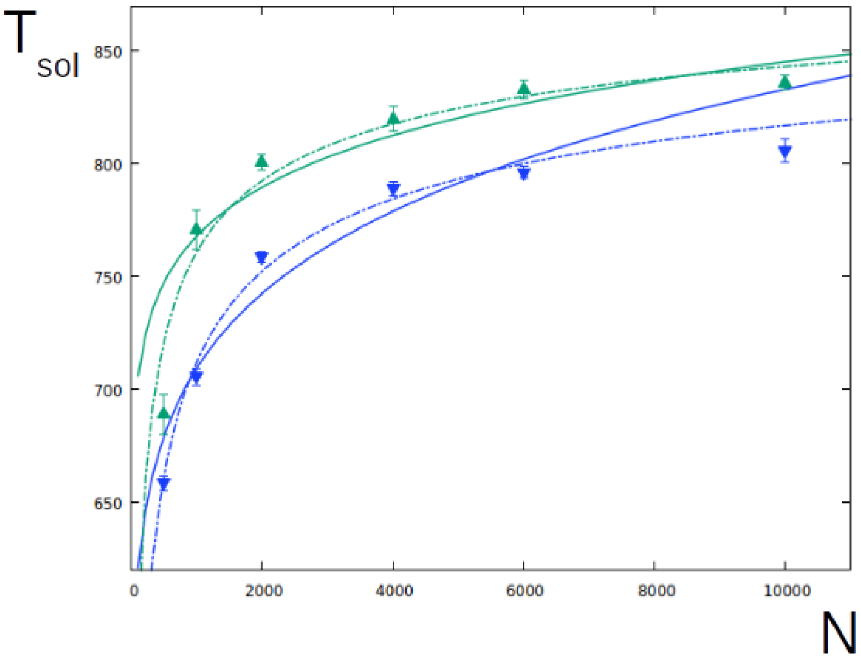

Figure S 5: Comparison of Eq. (S5) (solid lines) and Eq. (S4) (dash-dotted lines) with the simulation data for the final solidification temperature T_{sol} for cooling rate of 1 K/ns. Blue lines and symbols correspond to AgCo whereas green lines and symbols correspond to AgNi. The error bars on the symbols correspond to one standard deviation of the average T_{sol} over 10 independent simulations. Temperatures are in K.

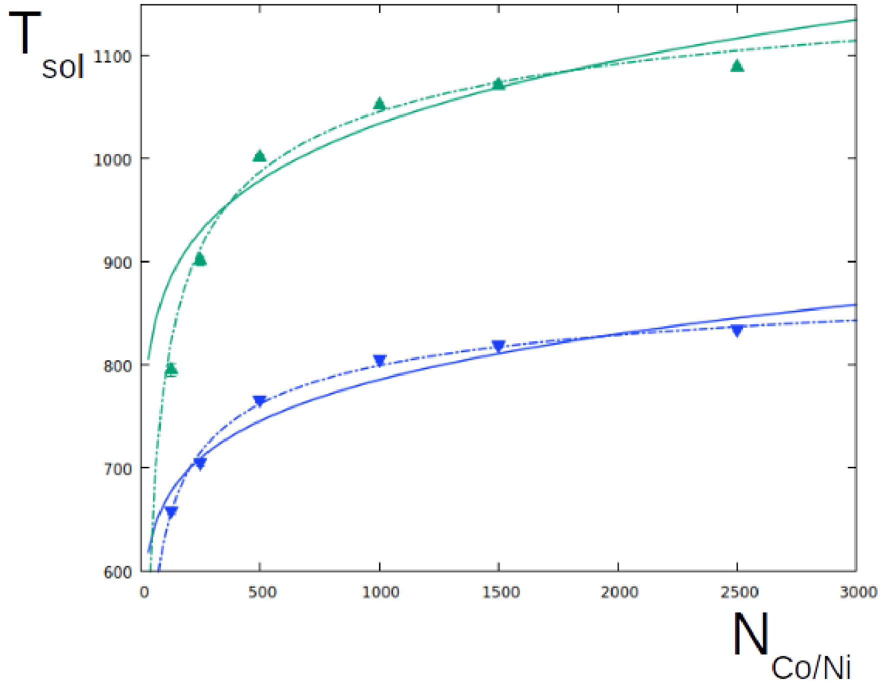

Figure S 6: Comparison of Eq. (S5) (solid lines) and Eq. (S4) (dash-dotted lines) with the simulation data for the solidification temperature T_{sol} of the Co-rich and Ni-rich parts for cooling rate of 1 K/ns. The data are reported as a function of the number $N_{Co/Ni}$ of Co or Ni atoms. Blue lines and symbols correspond to AgCo whereas green lines and symbols correspond to AgNi. The error bars on the symbols correspond to one standard deviation of the average T_{sol} over 10 independent simulations. Temperatures are in K. We note that for AgNi, the Ni-rich part solidifies at higher temperatures than the Ag-rich part for all sizes, whereas in AgCo separate solidification (i.e. the two-step pathway) occurs only for $N_{Co} \geq 500$ ($N \geq 2000$).

References

(1) Nelli, D.; Rossi, G.; Wang, Z.; Palmer, R. E.; Ferrando, R. Structure and orientation effects in the coalescence of Au clusters. *Nanoscale* **2020**, *12*, 7688–7699.

(2) Baletto, F.; Ferrando, R. Structural properties of nanoclusters: Energetic, thermodynamic, and kinetic effects. *Rev. Mod. Phys.* **2005**, *77*, 371–423.

(3) Bochicchio, D.; Ferrando, R. Morphological instability of core-shell metallic nanoparticles. *Phys. Rev. B* **2013**, *87*, 165435.

(4) Turnbull, D. Formation of Crystal Nuclei in Liquid Metals. *J. Appl. Phys.* **1950**, *21*, 1022–1028.

(5) Pawlow, P. N. Uber die Abhangigkeit des Schmelzpunktes von der Oberflachenenergie eines festen Korpers. *Z. Phys. Chem.* **1909**, *65*, 1–35.

(6) Sheng, H.; Lu, K.; Ma, E. Melting and freezing behavior of embedded nanoparticles in ball-milled Al–10wt% M (M=In, Sn, Bi, Cd, Pb) mixtures. *Acta Materialia* **1998**, *46*, 5195–5205.
